# Retrospective review of a tertiary adult burn centre’s experience with modified Meek grafting

**DOI:** 10.1186/s41038-016-0031-2

**Published:** 2016-02-26

**Authors:** Namal Munasinghe, Jason Wasiak, Andrew Ives, Heather Cleland, Cheng Hean Lo

**Affiliations:** 1 Victorian Adult Burns Service, The Alfred, 55 Commercial Road, Melbourne, 3004 Australia; 2 Epworth Hospital, 89 Bridge Road, Richmond, 3121 Australia; 3 School of Public Health & Preventive Medicine, Monash University, The Alfred, 55 Commercial Road, Melbourne, 3004 Australia

**Keywords:** Meek grafting, Postage stamp grafting, Micro-grafting, Massive burns

## Abstract

**Background:**

Autologous split skin grafting is the gold standard in treating patients with massive burns. However, the limited availability of donor sites remains a problem. The aim of this study is to present our experience with the modified Meek technique of grafting, outcomes achieved and recommendations for optimized outcomes.

**Methods:**

We retrospectively reviewed patient records from our tertiary referral burn centre and the Bi-National Burns Registry to identify all patients who had modified Meek grafting between 2010 and 2013. Patient records were reviewed individually and information regarding patient demographics, mechanism of injury and surgical management was recorded. Outcome measures including graft take rate, requirement for further surgery and complications were also recorded.

**Results:**

Eleven patients had modified Meek grafting procedures. The average age of patients was 46 years old (range 23 – 64). The average total body surface area (TBSA) burnt was 56.75 % (range 20–80 %). On average, 87 % of the grafted areas healed well and did not require regrafting. In the regrafted areas, infection was the leading cause of graft failure.

**Conclusions:**

Modified Meek grafting is a useful method of skin expansion. Similar to any other grafting technique, infection needs to be sought and treated promptly. It is recommended for larger burns where donor sites are not adequate or where it is desirable to limit their extent.

## Background

Early excision and wound closure with autologous split skin graft (SSG) of patients with severe burn injuries have been in use since the early 1970s [1, 2]. Whilst this is the standard of care in most burn centers [3], split skin grafting may be limited by donor site availability [4]. To overcome this clinical barrier, a number of techniques have been developed to allow for skin graft expansion including mesh grafting or micrografting [5].

CP Meek first described his technique in 1958 [6]. Harvested SSGs were expanded with a customized mesher to achieve the desired expansion. In 1964, Tanner introduced the mesh skin grafting technique commonplace nowadays [7]. The mesh grafting technique was user friendly and as a consequence developed rapid popularity while the Meek grafting technique fell out of favour. However Tanner’s mesh grafting technique was associated with lower expansion ratios and donor site availability remained a problem with patients with larger burns. In 1993, Kreis et al. modified Meek’s technique by using an updated air-compressed dermatome and incorporation of an aluminium foil backing [8]. Only recently has the modified Meek technique undergone a revival in the clinical setting [9].

The aim of this study was to review our experience with the modified Meek technique at an adult tertiary referral burn centre. In particular, the review’s intent was to describe our clinical protocol, determine patient outcomes and propose a series of recommendations for the successful use of the technique.

## Methods

### Study design

A retrospective chart review was performed on all patients admitted to our Unit from January 2010 to December 2013 who underwent wound closure with the modified Meek technique following severe burn injury. Patient records were manually reviewed; data collected include patient demographics, mechanism of injury, burn total body surface area (% TBSA), length of hospital stay, TBSA closed with the modified Meek technique, rate of graft take (%), microbiology results, episodes of repeat grafting required and outcomes achieved.

### Setting

The state of Victoria, Australia, has a population of 5.62 million people. Over two thirds of the population (73 %) live in the city of Melbourne. Our Unit is the statewide adult burns service, which also provides one of two designated major trauma services for adults in the state. Our Unit treats approximately 250 patients with acute burns each year.

### Ethics

Approval was obtained from our Health Human Research & Ethics Committee (project number 277/14).

### Surgical protocol/ guidelines

#### Debridement

All patients underwent early initial debridement using tangential excision, usually within 24 h after admission [1]. Within 72 h after initial debridement, debrided burn wounds were reviewed again in the operating theatre, with further debridement being undertaken, if necessary.

#### Microbiology

Intravenous cephazolin were used empirically preoperatively. If the history or microbiology suggested likely gram negative contamination, broad spectrum perioperative antibiotics were administered. Swabs were taken for microbiology at every dressing change and/ or trip to the operating theatre.

#### Wound closure

Immediate wound closure was undertaken using autologous meshed SSGs unless contraindicated by wound infection, uncertainty around wound depth, limited donor sites or patient instability. Excised ungrafted wounds were covered temporarily with either Biobrane® or cadaveric allograft.

#### Meek grafting

**Fig. 1:**
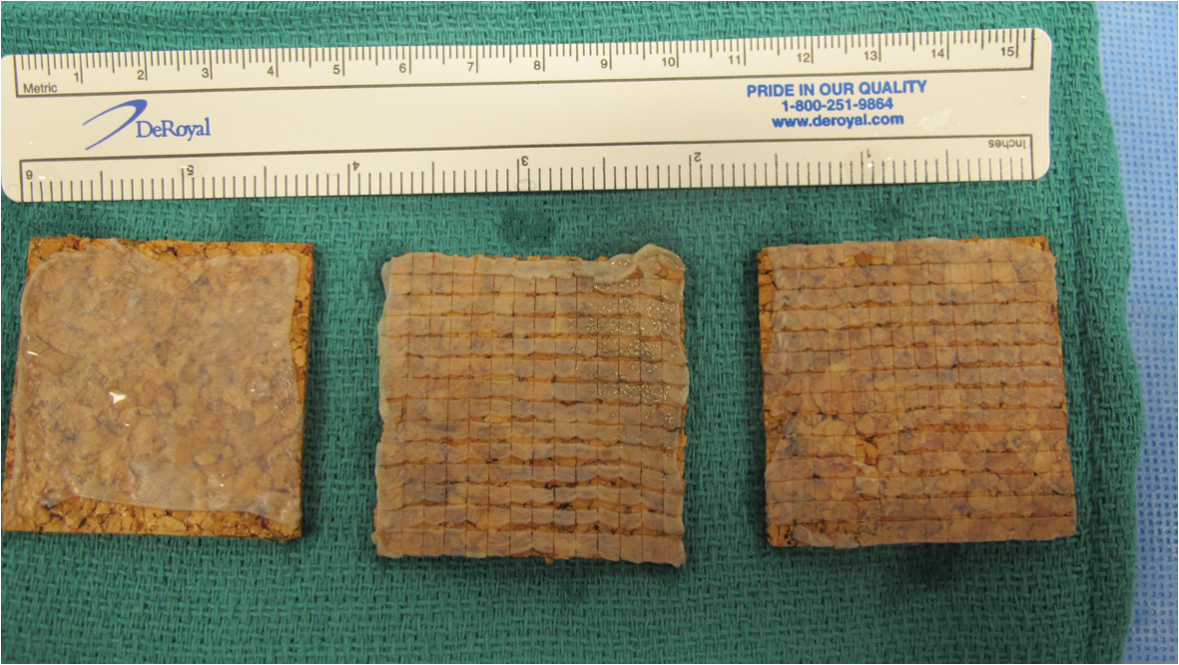
Split skin graft placed on the cork plate (dermal surface facing cork plate). Note that two of the plates (centre, right) had been passed through the mesher and is now ready for application onto the gauze for expansion

**Fig. 2:**
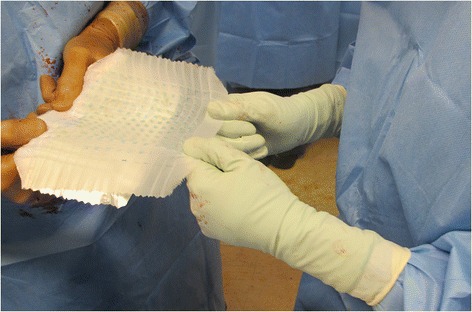
Expansion of small island grafts on a backing gauze

The surgical technique of modified Meek grafting has been previously described elsewhere (Fig. 1) [8, 10]. SSGs were harvested using a hand held Zimmer® dermatome, placed on a Meek cork board (dermal side down) and processed using the Humeca® Meek system. From a single 42x42 mm piece of split skin graft, 196 small island grafts were produced, to be expanded via a backing gauze that also serves to hold and fix the grafts onto the debrided wound bed (Fig. 2).

#### Dressings

Following initial debridement, burn wounds were dressed with silver-impregnated anti-microbial dressing Acticoat®, wrapped with moistened gauze and crepe bandages. Meek grafts were covered with moistened Acticoat® and gauze; the outer dressings were replaced every 3–5 days while the gauze backing left intact for approximately ten days.

### Statistical analysis

Because of the small number of subjects, non-parametric Spearman’s rank-order correlation was used to ascertain relationships between variables without correction for multiple comparisons. All comparisons used a *P* < 0.05 as indicating significance. Statistical analyses were performed using Prism version 6.0 for Mac OS X.

## Results

### Patient characteristic and burn injury profile

Over the four-year study period, 11 patients had wound closure using the modified Meek grafting technique in 12 operative procedures (data summarized in Tables 1 & 2). There were 7 male and 4 female patients, mostly middle aged (mean 46 years, range 18–77 years). The average burn TBSA was 56 % (range 20–85 %) and were all deep dermal or full thickness in depth and thermal related. These patients spent an average of 30 days in the intensive care unit (range 5–99 days) and the hospital length of stay was 98 days (range 44–167 days). There were no deaths.

**Table 1:** Baseline characteristics and injury profile (*n* = 11)

Gender	
Male	7
Female	4
Age (years)	46 (18–77)
TBSA burn (%)	56 (20–85)
ICU length of stay (days)	30 (5–99)
Hospital length of stay (days)	98 (44–167)

Data is average (range) or number. TBSA: total body surface area

**Table 2:** Details of each patient who received Meek grafts

Patient No.	Age (years)	Gender	TBSA burn (%)	Region	TBSA receiving Meek grafts (%)	Take rate (%)	Microbiology
1	39	Male	78	Back	8	100	none
2	64	Female	20	Lower limbs	12	33	Pseudomonas, Candida
3	47	Male	41	Back	18	72	Acinetobacter
4	52	Male	60	Flank/chest/ Lower limb	4	25	Pseudomonas, Candida
5	58	Female	55	Back/ abdomen/ upper limb	18	100	none
6	36	Male	85	Back/ abdomen/ upper limb	32	100	none
7	23	Male	75	Back/ chest/ abdomen	35	86	Enterobacter
8	49	Female	40	Flanks/ shoulder	5	100	MRSA
9	77	Male	38	Back	14	100	none
10	46	Male	60	Back	12	83	Pseudomonas
11	18	Female	63	Back	18	100	MRSA

TBSA: total body surface area; MRSA: methicillin-resistant Staphylococcus aureus

TBSA: total body surface area; MRSA: methicillin-resistant Staphylococcus aureus.

Statistically, wound infection reduced graft take (Spearman correlation −0.6517, *P* < 0.0001). The relationship between several other variables and outcome measures such as graft take rates and re-operation are summarized in Table 3.

**Table 3:** Summary of statistical analyses

Primary variable	Outcome measures	Spearman ρ	*P* value
Age	Graft take (%)	−0.2231	0.3458
Age	Re-operation*	0.0577	0.7922
TBSA burn (%)	Graft take (%)	0.2683	0.4191
TBSA burn (%)	Re-operation	−0.1736	0.3463
Anatomical region	Graft take (%)	−0.3057	0.2641
Anatomical region	Re-operation	0.1942	0.7835
TBSA Meek (%)	Graft take (%)	0.2006	0.5482
TBSA Meek (%)	Re-operation	0.0000	0.6970
Wound infection	Graft take (%)	−0.6517	<0.0001
Wound infection	Re-operation	0.6901	0.0909

*Re-operation refers to those patients who required repeat debridement and grafting of the areas which had previously received Meek grafting. TBSA: total body surface area

### Meek grafting technique

Meek grafts were most commonly used for the posterior trunk (67 %; 8/12 procedures) along with other anatomical regions including the lower limbs, anterior trunk and upper limbs. All patients received Meek grafts with 1:9 expansion ratios while one patient received both 1:9 and 1:6 expanded grafts. On average, 16 % (4–35 %) TBSA of each patient received Meek grafts.

**Fig. 3:**
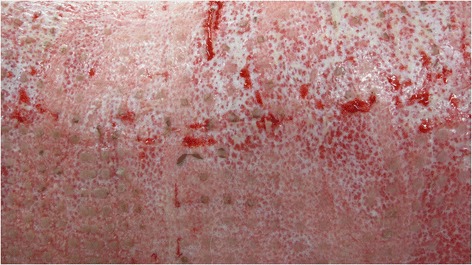
Wound bed with 3x3 mm islands of skin graft take in the early post-operative period

**Fig. 4:**
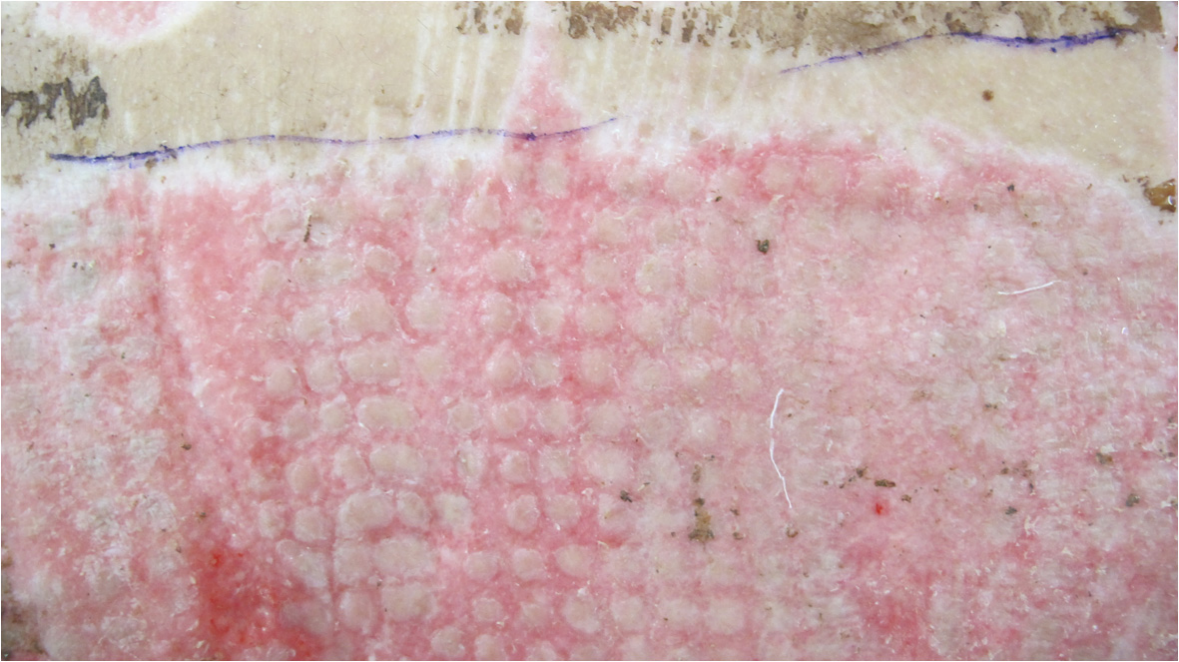
Wound bed almost completely closed following re-epithelialisation and healing by secondary intention between islands of skin grafts

In the eleven patients, modified Meek grafting achieved an average take rate of 87 % (25–100 %) (Table 2, Figs. 3 & 4). Five patients had partial graft failures ranging from 2–5 % TBSA requiring repeat grafting. Seven patients had positive swab cultures from Meek grafted areas. These organisms were predominantly Pseudomonas, Acinetobacter, Enterobacter and Candida species as well as methicillin-resistant *Staphylococcus aureus* (MRSA).

## Discussion

The average graft take rate of 87 % is comparable with the published literature [5, 8, 10–12]. Graft take was a clinical assessment of the areas healed or epithelialised, performed at removal of the gauze backing at the first comprehensive dressing change.

Statistical analyses revealed no significant associations between age and our outcome measures recorded (Table 3). It suggests that older patients may do equally well with Meek grafting while minimizing donor sites, in comparison to other grafting techniques with lesser expansion ratios and higher donor site morbidity. Our preference is to apply Meek grafts to burn wounds on the back; however these analyses indicate that Meek grafts survive equally well regardless of the anatomical region.

No significant associations were seen between the TBSA Meek grafts applied and the outcome measures studied. Increased Meek grafting did not decrease take rates or increase hospital length of stay, despite having higher expansion ratios and relying more on re-epithelialisation and healing by secondary intention. Statistical analyses also confirmed prior knowledge, that wound infection leads to poorer outcomes, including poorer graft take rates (Spearman correlation −0.6517, *P* < 0.0001).

In our institution, Meek grafting was most often applied to the back, followed by other sites including anterior trunk, upper and lower limbs. Our preference is to apply Meek grafts onto a wound bed with viable dermis. It is often possible to preserve viable dermis on the back, due to the thicker dermis in this region and mechanisms of injury. In this patient series, tangential excision sufficed while escharectomy is reserved for very deep burns where it is necessary to excise all ischaemic fat.

Meek grafting relies to a greater extent on re-epithelialization and therefore is to be avoided across joints, hands and head and neck regions to minimize contractures. Zermani et al. employed Meek grafts on regions with greater skin thickness, i.e. back, shoulders and hips with the belief that the thicker skin allowed greater conservation of pilosebaceous units and augmented healing [12].

Despite two thirds of our patients having had positive findings on wound microbiological assessments, our average Meek graft take rate remained at 87 %, comparable to figures from published series ranging from 85–95 % (see Table 4). None of our patients suffered complete graft loss. We concur with other published authors who found Meek grafts to be more resistant, or less compromised by infection when compared to meshed SSGs [5, 8, 10, 12].

**Table 4:** Summary of largest published series of Meek grafting in the English literature

Year	Author	Patients	Graft take (%)	Timing of assessment (days)^a^
1993	Kries et al. [8]	10	92	7
1997	Zermani et al. [12]	5	93	6
2001	Lari et al. [11]	7	90	7
2008	Hsieh et al. [5]	37	90 – 95	10
2009	Lumenta et al. [10]	6	85	10

^a^The number of days after grafting when assessments of graft take were performed

The outer dressings were replaced every 3–5 days whereas the backing gauze removed only after ten days to ensure graft adherence, prevent inadvertent graft removal with the backing and to optimize graft take. During these dressing changes it is often possible to detect the occurrence of infection under the gauze as it tends to lift off. If the gauze detaches or is easily removed then this is done; if the gauze were firmly adherent and dry then it is left in situ to avoid damage to regenerating epithelium. Other published authors removed the gauze and assessed graft take as early as 4–7 days after graft application and replaced the dressings with cadaveric allografts [11, 13]. In our experience, this measure was not necessary and as Lumenta et al. reported, we find satisfactory healing rates with Meek grafts alone [10].

**Fig. 5:**
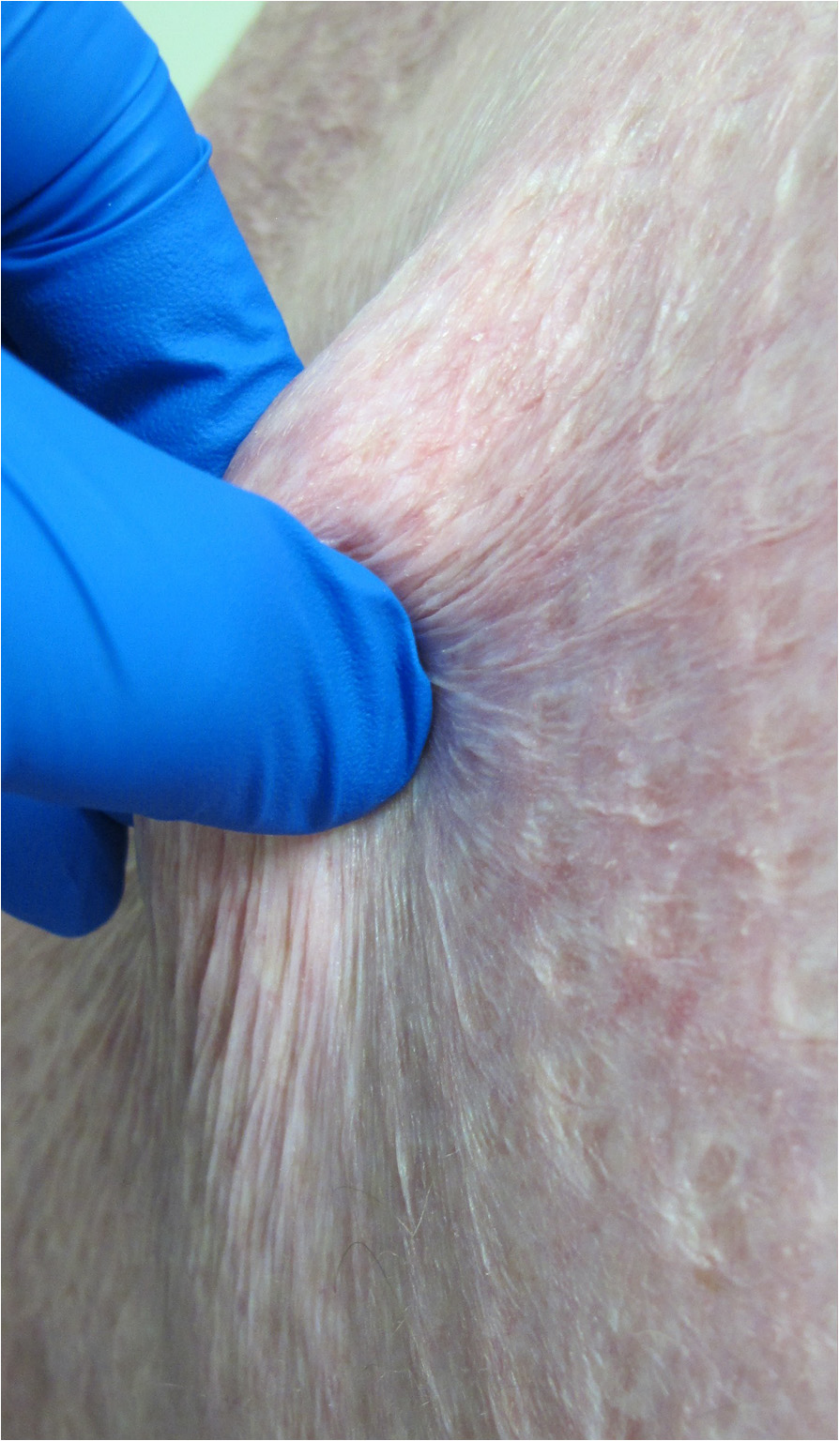
Long term outcome of Meek grafting with good tissue pliability

**Fig. 6:**
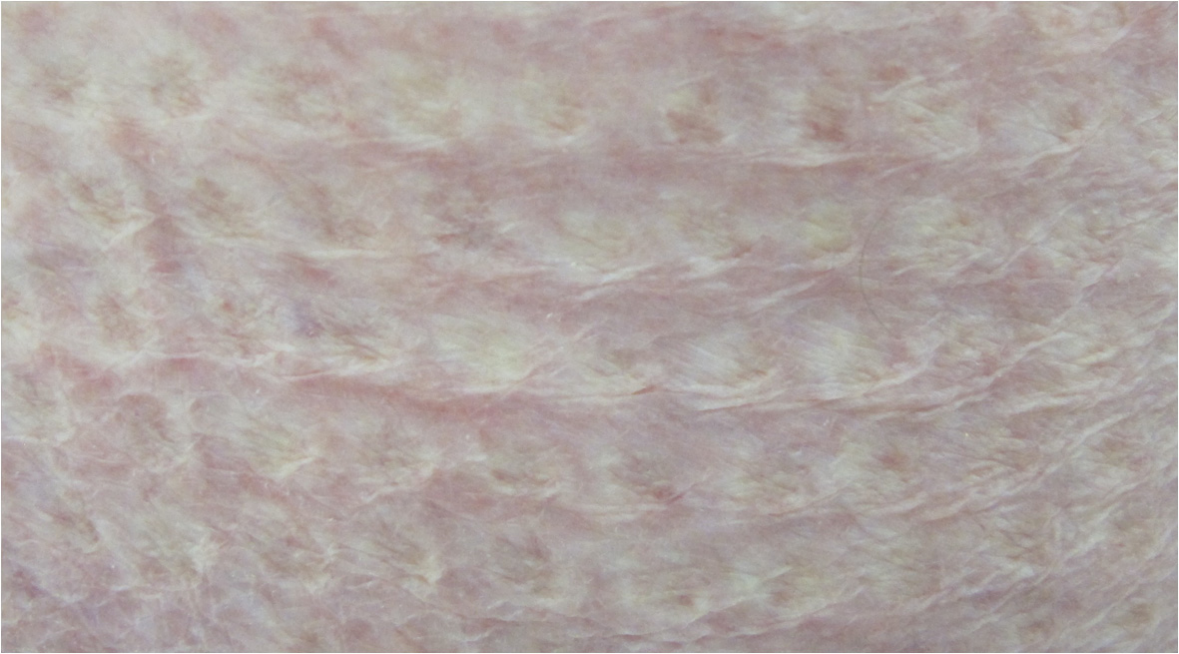
Long term outcome of Meek grafting with satisfactory cosmesis

This series is one of the largest published in the literature (Table 4) yet it is limited by its small sample size and retrospective nature. We have not focused on the long term results. However, our application of the modified Meek technique did not seem associated with long term problems such as poor cosmesis, scarring or joint contractures (Fig. 5). We agree with published series that cosmesis is comparable to widely expanded traditional meshed grafts (Fig. 6) [12].

## Conclusions

In conclusion, we find modified Meek grafting a reliable and useful technique in patients with limited donor sites. It is a relatively efficient technique with higher expansion ratio. Our overall Meek graft take rate of 87 % is consistent with the published literature. We recommend its addition to the armamentarium of all Burn Units.

## Abbreviations

SSG: split skin graft
TBSA: total body surface area
VABS: Victorian adult burns service
